# The Type 3 Deiodinase: Epigenetic Control of Brain Thyroid Hormone Action and Neurological Function

**DOI:** 10.3390/ijms19061804

**Published:** 2018-06-19

**Authors:** Arturo Hernandez, J. Patrizia Stohn

**Affiliations:** 1 Center for Molecular Medicine, Maine Medical Center Research Institute, Maine Medical Center, Scarborough, ME 04074, USA; stohnp@mmc.org; 2 Graduate School for Biomedical Science and Engineering, University of Maine, Orono, ME 04469, USA; 3 Department of Medicine, Tufts University School of Medicine, Boston, MA 02111, USA

**Keywords:** thyroid hormone, type 3 deiodinase, *Dio3*, environmental factors, *Dlk1*-*Dio3* genomic imprinting, behavior, brain development, sensory function, neuroendocrine function, brain morphology

## Abstract

Thyroid hormones (THs) influence multiple processes in the developing and adult central nervous system, and their local availability needs to be maintained at levels that are tailored to the requirements of their biological targets. The local complement of TH transporters, deiodinase enzymes, and receptors is critical to ensure specific levels of TH action in neural cells. The type 3 iodothyronine deiodinase (DIO3) inactivates THs and is highly present in the developing and adult brain, where it limits their availability and action. DIO3 deficiency in mice results in a host of neurodevelopmental and behavioral abnormalities, demonstrating the deleterious effects of TH excess, and revealing the critical role of DIO3 in the regulation of TH action in the brain. The fact the *Dio3* is an imprinted gene and that its allelic expression pattern varies across brain regions and during development introduces an additional level of control to deliver specific levels of hormone action in the central nervous system (CNS). The sensitive epigenetic nature of the mechanisms controlling the genomic imprinting of *Dio3* renders brain TH action particularly susceptible to disruption due to exogenous treatments and environmental exposures, with potential implications for the etiology of human neurodevelopmental disorders.

## 1. Introduction

The importance of thyroid hormones (THs) for brain development and function has been well established. Insufficient levels of TH during development due to congenital thyroid defects or to iodine deficiency may lead to impaired cognition and, in severe cases, to cretinism, a syndrome characterized by impaired sensory function, motor deficits, and profound mental retardation [1,2]. Studies in rodents have shown that THs regulate the expression of a large number of genes in the central nervous system (CNS) [3,4,5], affecting important neurological processes including myelination, synaptic establishment and transmission, dendrite formation, neuronal migration and maturation, axonal development, and the proliferation, fate and differentiation of neural cells [6,7,8]. Given the breadth of cellular and molecular processes influenced by THs in the CNS, it is not surprising that a disruption in the mechanisms controlling the action of THs leads to many neurological abnormalities affecting brain cytoarchitecture, motor, cognition and sensory functions, and behavior [9].

Circulating levels of THs largely depend on the regulation of the hypothalamic-pituitary-thyroid axis and the hormonal output of the thyroid gland. Levels of TH action in some tissues tend to correlate with serum hormone levels. However, a large body of research performed in recent years has dramatically increased our appreciation of the critical importance of the factors that regulate TH economy and action at the cellular level [10]. THs transporters [11] and metabolizing enzymes [12] may act at a local level to enhance or dampen TH action. This is critically applicable to the CNS, a tissue that exhibits a complex complement of these factors that uniquely regulate the trafficking and availability of TH in neural tissue. Thus, due to these local factors, serum levels of THs do not necessarily reflect TH action in a given tissue or target cell. This divergence between circulating level of TH and tissue TH action is particularly important in the context of the clinical evaluation of neurological disorders, as serum thyroid function parameters may not be indicative of the actual TH state of brain tissue.

The severity of neurological phenotypes resulting from congenital hypothyroidism directed most past scientific attention to the pathways increasing TH action and the positive effects of TH for neurological outcomes. However, significant evidence indicates that excessive TH action also has detrimental effects for brain development and function. In this regard, the predominant factor protecting the CNS from abnormally elevated levels of THs is the type 3 deiodinase (DIO3) [13]. The strong expression of DIO3 in the developing and adult brain suggests an important role for this enzyme in preventing neurological abnormalities caused by excessive TH action in the brain. Studies in DIO3-deficient mice, reviewed here, largely confirm this notion.

In addition, *Dio3* is one of the few genes undergoing genomic imprinting [14,15], an epigenetic phenomenon involved in the regulation of allelic gene expression depending on the allele’s parental origin [16,17]. In the context of current paradigms, the present review also focuses on the imprinting of *Dio3* in brain tissue, and explores its potential significance for brain development and neurological disorders.

## 2. Thyroid Hormone Action in the Brain: Role of DIO3

### 2.1. Main Mechanism of TH Action

Two main hormones are produced by the thyroid gland: thyroxine (3,5,3′5′-tetraiodothyronine, T4), which is produced in higher quantities and considered mostly a pro-hormone, and 3,5,3′-triiodothyroinine (T3), which is secreted in lower amounts but is the most active form, as it exhibits a 10-fold higher affinity than T4 for nuclear TH receptors. Both hormones are largely bound to proteins in the serum and can be transported into target cells by different types of cell membrane transporters including monocarboxylate transporters, organic anion transporters, and other members of the solute carrier transporter family [18]. These transporters provide a first level of specificity for TH action, as they exhibit different affinities for T3 and T4, and their number may vary significantly across target issues [19,20].

Once inside a target cell, THs can be deiodinated by members of a family of selenoenzymes that include type 1, 2, and 3 deiodinases (DIO1, DIO2 and DIO3, respectively) [12]. Via outer-ring deiodination, both DIO1 and DIO2 can convert the prohormone T4 into the active hormone T3, and thus, increase TH signaling. In contrast, DIO3 can convert T4 and T3 into 3,3′,5′-triiodothyronine (reverse T3, rT3) and 3,3′-diiodothyronine (3,3′-T2), respectively, both of which have a negligible affinity for the TH nuclear receptors. Thus, deiodinase enzymes in target cells regulate T3 availability and provide another tissue-specific level for the regulation of TH action.

Lastly, the predominant mechanism for the biological action of THs involves the regulation of gene transcription through nuclear TH receptors. These molecules are DNA-binding proteins that act as transcription factors, and upon T3 binding, regulate gene expression [10,21]. Two genes encode for the two basic types of TH receptors: TH receptor alpha and beta (THRA and THRB, respectively). These two genes express several receptor isoforms by alternative splicing that confer a last level of specificity in TH action, as they show different affinities for response elements in target genes, determine protein interactions, and drive co-factor recruitment in the regulation of gene transcription [22].

### 2.2. Noncanonical Mechanisms of TH Action

In addition to the most prevalent mechanism of TH action described above, recent observations are identifying other molecular pathways by which THs may exert biological effects. These mechanisms may include direct binding of T4 to the nuclear receptor to regulate gene transcription and binding of THs and their derivatives to receptors in the cytoplasm and cell membrane to regulate other cell signaling pathways [23,24]. More research is needed to further define the in vivo relevance of these mechanisms to normal physiology, but they may also be affected by DIO3 function, as this enzyme modulates the availability of THs and their metabolites.

### 2.3. Determinants of TH Action in the Brain: Role of DIO3

**Figure 1:**
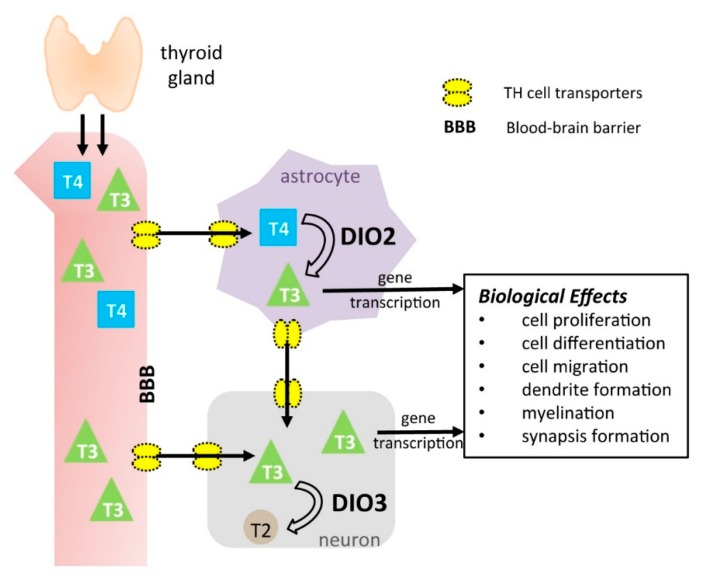
Mechanisms of thyroid hormone action in the brain and its biological effects. DIO2 and DIO3, type 2, and type 3 deiodinase, respectively. T3, triiodothyroinine; T2, diiodothyronine; T4, thyroxine.

Figure 1 shows the current working paradigm for TH action in the brain as supported by research in recent years. Transporters at the blood-brain barrier and in neural cell types are responsible for the transport of THs from the circulation into target cells. Among these transporters, the monocarboxylate transporter 8 (MCT8) and the organic anion carrier transporter 1c1 (OATP1C1) play predominant and critical roles in the brain transport of T3 and T4, respectively [25]. In humans, a genetic impairment in MCT8 function leads to the Allan-Herndon-Dudley syndrome, which features a severe hypothyroid state in the brain, and severe motor and cognitive defects similar to those observed in cretinism [26].

Also contributing to the TH economy in the neural milieu are the DIO2 and DIO3, which are predominantly expressed in astrocytes and neurons, respectively [27,28,29]. Despite their relatively high cell specificity, both enzymes can influence local hormone availability and T3-dependent gene expression not only in the cells in which they are expressed, but also in neighboring cells [30,31,32].

## 3. Consequences of DIO3-Deficiency for Brain Development and Function

### 3.1. Serum TH Status and Dio3 Expression in Development

THs are critical for normal brain development. However, their serum levels are very low during fetal life, not reaching adult-like levels until near birth in humans and two weeks of age in rodents. These periods are comparable in both species in terms of brain development milestones. During early development, serum THs can be lower than 5% of the adult values [33,34]. This period coincides with high expression of *Dio3* in most fetal tissues [35,36]. In addition, *Dio3* is present in mouse embryonic stem cells [37] and is highly expressed in the placenta [38,39] and in the maternal decidual tissue that surrounds the embryo after implantation [40]. The expression pattern of *Dio3* suggests that it is critical to limit TH action in early development, and that *Dio3* plays a crucial role in this regard.

For insight about the consequences of DIO3 deficiency for brain TH action, we need to consider how serum levels of TH and tissue *Dio3* expression change during development. In contrast to most mouse tissues whose *Dio3* expression is high during fetal and neonatal life and negligible in adulthood, the CNS exhibits high *Dio3* expression throughout life [27,41]. However, there are significant changes in *Dio3* expression levels among different brain regions and developmental stages [37,41]. During development, DIO3 activity is relatively high in the cerebellum, hindbrain, pons, and medulla, but declines to low levels in late neonatal life and adulthood. In contrast, the high DIO3 activity in the developing olfactory bulb, cerebral cortex, hippocampus, thalamus, hypothalamus, and striatum tends to be maintained into adult age, and could be even higher, especially in the cerebral cortex and the hippocampus [37]. Interestingly DIO3 activity peaks in the hypothalamus in early neonatal life [28,42], when most neuroendocrine systems are maturing. Marked, transient peaks in *Dio3* expression have also been described in the neonatal rat in specific neural structures related to brain sexual differentiation and the reward and fear systems including the amygdala, the nucleus accumbens, and the medial preoptic area [28]. Thus, DIO3 influences TH signaling in broad areas of the developing and adult brain, protecting neural processes from untimely or excessive T3 action, and ultimately ensuring normal CNS function in adulthood.

### 3.2. Neurological Phenotypes of Mice Lacking DIO3 

**Figure 2:**
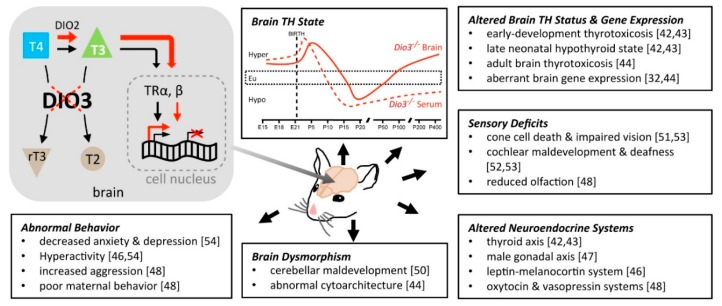
Neurological consequences of DIO3 deficiency in mice. The lack of DIO3 function prevents degradation of THs, increasing their availability and molecular action in the brain (red lines and arrows). Increased T3 action in the brain (grey arrow) leads to multiple neurological phenotypes (black arrows). TR, thyroid receptor; DIO2, type 2 deiodinase.

The importance of DIO3 for the CNS is evidenced by observations in mice carrying a mutation that renders DIO3 fully inactive. *Dio3^−/−^* mice exhibit an array of neurological abnormalities, as described below and summarized in Figure 2.

#### 3.2.1. TH Status of the *Dio3^−/−^* Brain

In wild type (WT) mice, serum levels of THs peak and reach adult-like levels at 2–3 weeks of age. Loss of DIO3 function translates into severely impaired clearance of THs during fetal and neonatal life. As a result, *Dio3^−/−^* mice experience developmental thyrotoxicosis, characterized by high serum levels of T3 and low levels of serum T4 (Figure 2) due to T3 negative feedback on the hypothalamic-pituitary-thyroid (HPT) axis [42]. This thyrotoxicosis occurs during the time the HPT axis is maturing physiologically. However, due to the T3-driven suppression of the HPT axis, *Dio3^−/−^* mice at later developmental stages are hypothyroid, exhibiting lower than normal serum levels of T3 and T4 (Figure 2) [42]. Although the HPT axis functional deficits of *Dio3^−/−^* mice ameliorate in adulthood, they never fully recover and *Dio3^−/−^* mice exhibit low serum levels of T3 and T4 during adult life and substantial impairments in the regulation of the axis [43].

Based on the assessment of T3-dependent gene expression, the brain of *Dio3^−/−^* mice largely follows the TH state of the serum, being thyrotoxic in early development and hypothyroid in late neonatal life (Figure 2) [42]. However, the adult *Dio3^−/−^* brain becomes increasingly thyrotoxic with age, despite the serum hypothyroidism. Notably, these age-dependent changes in brain TH status do not take place in the same time frame across the CNS. Different regions of the brain show specific timelines for reaching a hypothyroid state during late neonatal life and a hyperthyroid state in adulthood [44]. This is likely the result of regional differences in the molecular determinants of TH action at the local level. Notably, the T3 excess in the *Dio3^−/−^* brain is largely normalized with concurrent DIO2 deficiency [45], illustrating the important and complementary role of these enzymes in maintaining brain TH action within an adequate range.

The divergence between the TH states of the serum and the brain that occurs in *Dio3^−/−^* mice is of critical importance in the clinical context of neurological disorders. It raises the possibility that a deficiency in DIO3 may lead to an excess of T3 in the brain that is not appreciated by evaluating thyroid parameters in the serum.

#### 3.2.2. Neuroendocrine Abnormalities

In addition to the functional deficits in the HPT axis mentioned above, *Dio3^−/−^* mice also manifest abnormalities in the leptin-melanocortin system, which controls energy balance by regulating food intake and energy expenditure [46]. The hypothalamus of *Dio3^−/−^* mice exhibits increased expression of agouti-related protein and decreased expression of pro-opiomelanocortin [46]. This is observed together with serum leptin levels that are normal or high. These parameters would normally be associated with increased food intake, and reduced energy expenditure and leptin resistance, and would predict an obesity phenotype. However, *Dio3^−/−^* mice are leaner and manifest reduced adiposity, likely due to a markedly increased level of physical activity [46].

*Dio3^−/−^* mice of both sexes manifest impaired fertility [42]; in the males this is associated with hormonal alterations in the gonadal axis [47], suggesting abnormalities in the reproductive functions of the endocrine hypothalamus.

Adult *Dio3^−/−^* mice also exhibit reduced serum levels of oxytocin (OXT) and arginine-vasopressin (AVP), and abnormal, sexually dimorphic gene expression patterns related to the signaling of these neuropeptides [48]. Serum OXT and AVP are reduced mostly in adult *Dio3^−/−^* females, while hypothalamic *Oxt* and *Avp* mRNA expression is largely affected in male *Dio3^−/−^* mice, being increased in neonates and reduced in adults [48]. These findings suggest hypothalamic T3 excess results in sexually dimorphic abnormalities in the physiology of these neuropeptide systems, which are highly relevant to social behaviors [49].

#### 3.2.3. Brain Morphology

The developmental T3 excess in *Dio3^−/−^* mice results in a brain with several morphological abnormalities, many of them not characterized in full (Martinez et al. unpublished observations). The *Dio3^−/−^* cerebellum is hypomorphic. It shows reduced foliation, accelerated disappearance of the external germinal layer, and premature expansion of the molecular layer at juvenile ages [50], abnormalities that are associated with impairments in motor tasks. This phenotype is normalized in a genetic background lacking THRA [50], suggesting that the aberrant cerebellar outcomes in *Dio3^−/−^* mice are caused by increased T3 signaling through this particular receptor.

#### 3.2.4. Sensory Function

DIO3 is highly expressed during development in the retina [51], middle ear [52] and olfactory bulb [37]. In this regard, *Dio3^−/−^* mice manifest substantial deficits in sensory function. The developmental excess of T3 in *Dio3^−/−^* mice disrupts cochlear development and leads to deafness [52]. It also causes the neonatal degeneration and death of retinal cones [51], which are critical for light and color vision. A concurrent loss of function in DIO2, the enzyme that enhances T3 availability (Figure 1) leads to a significant amelioration of the abnormal TH state in the serum [53] and brain [45] of DIO3-deficient mice, suggesting that DIO2 activity is exacerbating the thyrotoxicosis caused by impaired TH clearance by DIO3. Interestingly, in mice with double DIO2/DIO3 deficiency, cone viability is normalized, but the deafness is not only not eliminated, but is more profound [53]. The latter observation suggests that ear development requires strict and timely control of T3 action for normal outcomes, and best illustrates the idea that multiple alterations in the developmental pattern of TH action may have additive effects on brain pathophysiology. In addition, female *Dio3^−/−^* mice exhibit impaired olfactory function [48], although the molecular and cellular basis for this phenotype remains to be identified.

#### 3.2.5. Behavior

*Dio3^−/−^* mice exhibit alterations in behavior that are relevant to neurological conditions in humans. *Dio3^−/−^* mice of both sexes manifest hyperactivity and reduced anxiety-and depression-like behaviors [54]. *Dio3^−/−^* females exhibit poor maternal behavior, and mutants of both sexes show increased levels of threat and aggressive behaviors [48]. In addition, the hyperactivity in adult *Dio3^−/−^* mice of both sexes is associated with a lengthened circadian cycle of night activity [46].

In summary, the absence of DIO3 has broad consequences for the TH status of the brain and for TH-dependent programs of brain gene expression, ultimately affecting brain morphology, sensory and neuroendocrine functions, mood and social behaviors, physical activity and circadian patterns. There are no current cases of *DIO3* inactivating mutations described in humans. However, the above observations suggest that DIO3 deficiency may have important implications for neurodevelopmental and neurological disorders.

## 4. Genomic Imprinting of *Dio3*

### 4.1. Genomic Imprinting

The multiple effects of DIO3 deficiency on brain development and function provides important relevance to the mechanisms regulating its expression. A critical mechanism is genomic imprinting, an epigenetic phenomenon affecting a small percentage of genes that results in preferential or exclusive expression from one of the alleles, depending on the allele’s parental origin [17,55]. This allelic expression pattern is the result of sex-specific epigenetic marks (DNA methylation) in the gametes that are maintained after fertilization and during embryonic development [17,56], leading to allele-specific expression or repression of the imprinted gene [17,57,58]. Disruption of the mechanisms regulating genomic imprinting leads to aberrant dosages of imprinted genes, and results in pathological outcomes in humans and animal models [59,60,61,62].

### 4.2. The Dlk1-Dio3 Imprinted Domain

Imprinted genes are usually located in distinct clusters (“imprinted domains”) across the genome. Each cluster typically features one or more genomic regions exhibiting allele-specific differential methylation [63,64,65]. Some differentially methylated regions function as “imprinting control regions (ICRs)” and are responsible, depending on their methylation status, for directing the expression or repression of imprinted genes within the cluster in *cis* [17,66].

**Figure 3:**
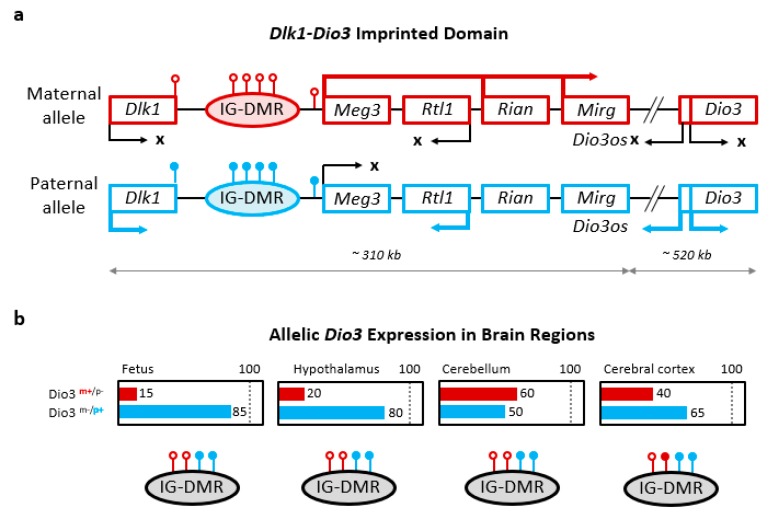
Genomic imprinting of *Dio3* in the brain. (**a**) Simplified diagram of the mouse *Dlk1-Dio3* imprinted domain showing the dominant pattern of allele-specific gene expression. An arbitrary number of pin point shapes indicate loci exhibiting allele-specific methylation (open circles, unmethylated; closed circles, methylated); (**b**) Brain variability in the percentage allelic contribution to *Dio3* expression and associated IG-DMR methylation compared to fetal *Dio3*. Some brain regions exhibit relaxed or absent *Dio3* imprinting despite unchanged IG-DMR methylation status [37]. (Data is approximate and based on parent-of-origin inheritance of the DIO3 mutation. Allelic contributions may add more than 100%, as the wild type allele may exhibit T3-dependent up-regulation upon loss of DIO3 function in the other allele).

*Dio3* belongs to what is usually referred to as the *Dlk1*-*Dio3* imprinted domain [67], which is defined by the genes, *Dlk1* and *Dio3*, that initially marked the centromeric and telomeric ends of the imprinted cluster in mouse chromosome 12 [68]. (Later, another imprinted gene, *Begain*, was described as located centromeric to *Dlk1* [69]) This domain is located in the distal arm of mouse chromosome 12 and the syntenic region in the distal arm of human chromosome 14 [70]. A simplified diagram of the mouse *Dlk1*-*Dio3* imprinted domain is shown in Figure 3a. It includes the *Dlk1*, *Rtl1,* and *Dio3* genes that are preferentially expressed from the paternal allele (“paternally expressed”) [14,15,71], and the *Meg3*, *Rian* and *Mirg* genes, which are preferentially expressed from the maternal allele (“maternally expressed”). Interestingly, while paternally expressed genes in the domain are protein-encoding, maternally expressed genes include different types of non-coding RNAs [71,72,73].

Allelic expression in imprinted domains is directed by allele-specific differential methylation. In the *Dlk1*-*Dio3* domain, three main regions have been identified as differentially methylated. These include the 3′ end of *Dlk1*, the promoter region of *Meg3*, and an intergenic, differentially methylated region (usually referred to as IG-DMR) located between the *Dlk1* and *Meg3* genes [65,74]. These regions are hypomethylated in the allele inherited from the mother and hypermethylated in the allele inherited from the father [71]. The lack of methylation in the maternal allele is associated with the expression of maternally expressed non-coding RNAs and the *in cis* repression of paternally expressed protein-coding genes (Figure 3a).

### 4.3. Regulation of Dlk1-Dio3 Genomic Imprinting

The IG-DMR functions as the ICR of the *Dlk1*-*Dio3* imprinted domain [75]. Maternal allele deletion of the IG-DMR leads to increased methylation at the *Meg3* promoter and subsequent repression of maternally-expressed genes. It also leads to aberrant expression of paternally-expressed genes from the maternal allele [65,76]. These genes, including *Dio3*, will then show biallelic expression.

In contrast, IG-DMR deletion in the paternal allele does not affect the expression of genes in the domain [65]. These observations show that the IG-DMR is critical for the control of the *Meg3* promoter and the expression of maternally-expressed genes, and suggest that the latter are needed for the normal repression of paternally-expressed genes in the maternal allele, including *Dio3* [76].

Consistent with this idea are observations in mouse models carrying a lacZ transgene insertion at the *Meg3* promoter region [77]. Mice with paternal or maternal inheritance of this transgene exhibit aberrant placental and fetal expression of the imprinted genes in the domain, and these changes are associated with abnormalities affecting viability, growth, and development [77,78].

### 4.4. Dio3 Genomic Imprinting Across Tissues

In the mouse fetus, *Dio3* exhibits a strong preferential expression from the paternal allele [14,15], but the maternally-inherited *Dio3* allele is not completely silenced [15]. Compared to other mouse fetal tissues, *Dio3* imprinting is markedly relaxed in the placenta [79]. However, the molecular basis for this relaxation is unknown. The different degree of imprinting in placental and fetal *Dio3* is consistent with the different response of these tissues to the disruption of the *Meg3* promoter region. A transgene insertion at the *Meg3* promoter in the paternal allele does not change *Dio3* expression in the placenta, but reduces it in the fetus [78]. In contrast, maternal inheritance of this insertion causes a marked increased in placental *Dio3* expression while leaving fetal *Dio3* expression unchanged [77]. These observations illustrate the functional role for maternally expressed genes in controlling *Dio3* expression, and suggest that there are important, unidentified intrinsic differences in how the imprinting of *Dio3* is regulated in the placenta and fetus. These differences may also apply to other tissues, including the developing testis and retina, which show biallelic *Dio3* expression [37].

*Dio3* imprinting variations across tissues and developmental stages are characteristics that are consistent with observations in other imprinted genes [17].

### 4.5. Dio3 Genomic Imprinting in the CNS

We have used a genetic model of DIO3 inactivation to assess allelic contributions to the overall levels of *Dio3* expression in brain regions. These studies showed that *Dio3* is imprinted in the fetal mouse brain, and preferentially expressed from the paternal allele. However, the contribution of the maternal allele to brain *Dio3* expression is not negligible [37]. In the fetal brain, preferential paternal *Dio3* expression is observed in the most abundant and well-characterized 2.2 kb *Dio3* transcript, as well as in larger, uncharacterized *Dio3* transcripts [37].

In the mouse newborn brain, the degree of preferential *Dio3* expression from the paternal allele varies significantly across brain regions (Figure 3b), being strongest in the hypothalamus and moderate in the cerebral cortex, hippocampus and striatum [37]. In the neonatal cerebellum, biallelic *Dio3* expression is observed. At weaning age, overall levels of *Dio3* expression decrease in many brain areas (except the cerebral cortex and hippocampus), and the degree of monoallelic *Dio3* expression tends to be further reduced [37].

This variability in imprinting is not necessarily associated with changes in the methylation status of the IG-DMR (Figure 3b). In the neonatal cerebral cortex and retina, increased IG-DMR methylation is associated with a reduced degree of monoallelic *Dio3* expression [37]. In these cases, the presumed gain of methylation in the maternal allele may explain an increased contribution of this allele to overall *Dio3* expression. However, in other brain regions including the cerebellum (and the placenta mentioned above), the reduced degree of monoallelic expression cannot be explained by gains of methylation in the IG-DMR [37,65].

Furthermore, observations in rats also indicate preferential *Dio3* expression from the paternal allele in the fetal brain, and a relaxation towards biallelic expression in the adult brain [80,81]. These studies also found variations in *Dio3* imprinting across brain regions. Interestingly, they reveal that the adult hippocampus exhibits preferential *Dio3* expression from the maternal allele [80,81]. Although not overt, this allelic expression pattern is not associated with changes in IG-DMR methylation status, again suggesting the existence of unidentified underlying mechanisms.

Most of the variations in allele-specific expression of *Dio3* across brain regions are accompanied by correlating changes in DIO3 activity [37], indicating that the imprinting status of a specific brain region may impact local T3 availability and action. However, there is insufficient data available about whether aberrant *Dio3* imprinting affects the expression of local T3 target genes, or which brain regions or developmental stages are more sensitive. In addition, in models of altered *Dio3* imprinting, developmental systemic levels of T3 are also altered [37], and may impact brain T3 responses. Thus, more work with suitable experimental models is needed in this regard.

It is worth noting that, in contrast to the well-characterized 2.2 kb transcript predominantly expressed during development, larger *Dio3* transcripts are more abundant in the normal adult brain [27]. Thus, these observations raise the possibility that the genomic imprinting of *Dio3* is not only tissue-specific, but also transcript-specific, a characteristic that has also been observed for other imprinted genes [82].

### 4.6. Other Genomic Elements in the Dio3 Gene Locus

Additional conserved genomic features close to the *Dio3* gene may be of functional significance to its expression. These include a long non-coding RNA, *Dio3os* (for *Dio3* opposite strand) and a conserved enhancer [83,84]. *Dio3os* is located head-to-head with the *Dio3* promoter region, and transcribes from the opposite strand multiple transcripts via alternative splicing [84]. *Dio3os* shows preferential monoallelic expression in multiple cattle tissues [85], but there is insufficient data about its imprinting status in mice and humans. *Dio3os* expression strongly correlates with that of *Dio3* in human cell lines [86], rat brown preadipocytes [87], rat brain [88], and mouse uterus [89]. The biological function of *Dio3os* is unclear, but given the overlap of the *Dio3* and *Dio3os* promoter regions, it is possible that *Dio3os* transcription modulates that of *Dio3*, as suggested by studies on mouse decidual tissue [89].

The enhancer is located 3′ of the *Dio3* gene, and features serum and AP1 response elements that are well conserved between species [90]. This enhancer is capable in vitro of transactivating the *Dio3* promoter [90] in response to serum and growth factors, but its functional significance in vivo has not been determined.

In view of the observations above, *Dio3* appears to be the only gene in the domain that does not exhibit strict imprinting, suggesting the existence of other unidentified epigenetic factors that influence *Dio3* imprinting in certain tissues, including the developing and adult brain. In addition, given that brain *Dio3* expression is largely located in neurons [27], and that these cells contribute only a minor proportion of the brain DNA pool, it is possible that overall methylation in brain tissue does not accurately reflect the methylation status of *Dio3*-expression cells. It is also possible that a high degree of *Dio3* imprinting in more restricted brain regions or neuronal types is not appreciated when larger brain areas are examined. Additional research is required to address this issue.

### 4.7. Genomic Imprinting of Human DIO3

The *Dlk1*-*Dio3* imprinted domain and their predominant patterns of allelic expression are highly conserved in humans [91] and multiple mammalian species [85,92,93,94,95].

In the human *DLK1*-*DIO3* imprinted domain, the pattern of allele-specific gene expression is largely conserved [67,96]. Human studies have suggested that *DIO3* is not imprinted in the placenta [97,98], an observation that is consistent with the relaxed placental imprinting of the mouse *Dio3* [79]. A recent study in foreskins from infants indicates that human *DIO3* is an imprinted gene, showing a strong pattern of preferential expression from the paternal allele [99] similar to that observed in the mouse fetus. In this human newborn tissue, *DLK1* is exclusively expressed from the paternal allele [99], consistent with previous studies in other human fetal tissues [96].

In addition, human *DIO3* was found to be preferentially expressed from the maternal allele in an adult skin biopsy [99]. Since in many adult human tissues—including the brain—larger *DIO3* transcripts are the most abundant [84], this observation is consistent with findings in the adult rat hippocampus [80], and with the untested hypothesis that larger *DIO3* transcripts, which are more abundant in the adult rodent brain and apparently expressed from an unidentified alternative promoter, exhibit preferential expression from the maternal allele in adulthood.

## 5. *Dio3* Imprinting in Brain Disease and Evolution

### 5.1. Altered Dlk1-Dio3 Imprinting in Mice and Humans

Many imprinted genes are highly expressed in the placenta and fetus and play critical roles in growth, development and behavior [62,100]. The importance of imprinting at the human *DLK1*-*DIO3* domain is evident from patients with Temple or Kagami-Ogata syndromes [101]. These syndromes are the result of aberrant imprinting due, respectively, to maternal or paternal uniparental disomy (UPD) of chromosome 14 (UPD14) [102], where the *DLK1*-*DIO3* domain is located [96]. These patients exhibit abnormal expression of the imprinted genes in the *DLK1*-*DIO3* domain, and may manifest growth retardation, craniofacial dysmorphisms, abnormal rib cages, altered puberty onset, hypotonia, hydrocephalus, and mental retardation [103,104].

Consistent abnormalities are observed in mouse models of altered *Dlk1*-*Dio3* imprinting. Paternal or maternal UPD12 impacts perinatal viability, placental and fetal growth, and skeletal development [105]. Mice carrying a deletion of the IG-DMR in the maternal allele exhibit comparable defects [79]. In addition, a transgene insertion at the *Meg3* promoter region disrupts allelic expression in the domain, and influences metabolic adaptation to independent life when maternally inherited [77], or disrupts growth, growth hormone axis physiology and adult metabolism when paternally inherited [78].

Despite the evidence demonstrating the deleterious consequences of altered *Dlk1*-*Dio3* gene dosage, it is difficult to discern the contributions of the individual genes in the domain to the abnormalities observed. Genetic mouse models that disrupt the allelic expression of specific genes may shed some light on this issue, although these mutations tend to be associated with aberrant expression of other genes in the domain, making it difficult to exclude secondary alterations in neighboring imprinted genes as the causes of the observed phenotypes. Concerning DIO3, although some phenotypes of mouse DIO3-deficiency are consistent with those of aberrant *Dlk1*-*Dio3* imprinting in mice and human syndromes, others are not or not known. Thus, the particular contribution of increase T3 action during development to the generation of these syndromes remains to be determined.

### 5.2. Altered Dlk1-Dio3 Imprinting and Brain Development and Function

If altered *Dio3* expression were partially responsible for the phenotypes caused by abnormal imprinting in the *Dlk1*-*Dio3* region, one would expect that some phenotypes of the *Dio3^−/−^* mouse will be partially consistent with the abnormalities observed in mouse and human models of abnormal *Dlk1*-*Dio3* imprinting that exhibit deficient expression of *Dio3*. In this regard, the impaired perinatal viability and growth retardation of *Dio3^−/−^* mice [42] are consistent with observations in mice with maternal UPD12 [105] and with the poor suckling behavior, failure to thrive, and stunted growth of infants with Temple syndrome [106].

Concerning neurological defects, no information is available from mice with maternal UPD12, since these animals die before reaching adulthood. However, the reduced cerebellum [50] and hydrocephalus [44] of *Dio3^−/−^* mice is consistent with the reduced head circumference and hydrocephalus observed in Temple syndrome patients [106]. Although these patients also exhibit mild mental retardation, no abnormalities have been reported in relation to anxiety, depression, aggressive behavior or hyperactivity, as those manifested by *Dio3^−/−^* mice.

Thus, DIO3 deficiency, and the excessive T3 action associated with it, may contribute specifically to the abnormalities caused by altered imprinting in the *Dlk1*-*Dio3* domain. Despite the tissue variability in *Dio3* imprinting, developmental T3 excess has been demonstrated in mouse models with no *Dio3* expression from the paternal allele [14,37]. It is thus possible that allele-specific inactivation of *Dio3* leads to neurological phenotypes, especially those dependent on brain regions exhibiting highest degree of monoallelic and overall *Dio3* expression.

Deficiencies in paternally expressed *Dio3* and in maternally expressed microRNAs in the imprinted domain seem to have opposite effects on neurological phenotypes. Deletion of the miR-379/miR-410 gene cluster at the imprinted domain enhances anxiety-related behavior [107], in contrast with the decreased anxiety-related behavior observed in *Dio3^−/−^* mice. Also, loss of non-coding RNA expression from the *DLK1-DIO3* imprinted locus correlates with reduced neural differentiation potential in human embryonic stem cell lines [108], while T3 signaling, which is increased in DIO3-deficiency, is known to enhance neurogenesis [109,110]. The role of the *Dlk1*-*Dio3* imprinted domain in neural cell homeostasis is further supported by the observation that a postnatal loss of *Dlk1* imprinting in stem cells and niche astrocytes regulates neurogenesis [111].

In addition, a microRNA signature associated with schizophrenia includes the down-regulation of 17 microRNAs expressed from the *Dlk1*-*Dio3* domain [112]. It is not uncertain whether other imprinted genes in the domain including *Dio3* may also be affected in this condition, but a relationship between *Dio3* and microRNAs in the domain has been observed. In a model of myocardial infarction, *Dio3* expression is associated with the induction of a pluripotency microRNA signature from the *Dlk1*-*Dio3* genomic region [113], suggesting a reciprocal association between the expression of paternal *Dio3* expression and maternal microRNAs.

The inverse relationship in the brain between the expression of *Dio3* and non-coding RNAs is further supported by their expression pattern in brain cells. Like *Dio3*, and according to data from cell-specific gene expression profiling in the cerebral cortex of the developing mouse [114], *Meg3, Rian and Mirg* are highly specific to neurons, suggesting that an adequate balance of paternal (*Dio3*) and maternal (non-coding RNAs) gene expression in the domain is necessary for adequate T3 action on this cell type.

### 5.3. Dio3 Imprinting and Environmental Factors

Due to their finely tuned epigenetic regulation, the expression of imprinted genes is particularly susceptible to environmental or exogenous factors [115]. Exposure to chemicals, diet, and stressors are but a few of the factors that can interfere with the epigenetic mechanisms that govern genomic imprinting [115].

Few factors have been identified to alter *Dio3* allelic expression. Ascorbic acid has been found to prevent loss of imprinting at the *Dlk1*-*Dio3* domain in stem cells [116,117], and maternal immune response or cannabinoid exposure in adolescence alters the imprinting of *Dlk1*-*Dio3* in the entorhinal cortex, a region implicated in schizophrenia [118].

An important body of work specifically concerning *Dio3* imprinting and environmental factors has been produced by the group of Eva Redei. Her laboratory has studied a rat model of fetal alcohol exposure using rat strains that carry a single nucleotide polymorphism in the *Dio3* exon, allowing for *Dio3* allele discrimination. Fetal alcohol exposure leads to an abnormal behavioral profile in adulthood of relevance to autistic spectrum disorders, including deficits in social behavior, anxiety and fear-induced memory [119,120,121]. This developmental insult also results in abnormal *Dio3* imprinting and subsequent increase of *Dio3* expression in the hippocampus, but not other brain regions such as the amygdala [121,122]. Part of the effects of alcohol exposure on behavior and *Dio3* expression can be modified by developmental treatment with T4 or metformin [120,121]. Notably, maternal alcohol exposure resulted in decreased fetal and placental *Dio3* expression [123]. Although these studies cannot discern the extent to which changes in brain *Dio3* expression contribute to the behavioral abnormalities caused by alcohol exposure, they demonstrate that this environmental factor, as well as T4 or metformin treatments, influences *Dio3* imprinting and subsequent TH action in the brain.

Fetal alcohol exposure also leads to behavioral phenotypes and abnormal *Dio3* imprinting in subsequent F1 and F2 generations [119,120]. These effects can be corrected by thyroxine treatment, and are sexually dimorphic and dependent on parental lineage. For instance, fetal alcohol exposure increases anxiety related behavior in F1 generation males, but decreases it in corresponding females [119]. In this model it is possible that other genomic *loci* of relevance to the CNS function are also affected and contribute to the behavioral phenotypes. However, these findings show that *Dio3* imprinting and expression are susceptible to alcohol exposure in a manner that can be inherited via epigenetic mechanisms, and that interventions in future generations (e.g., treatment with T4 or metformin) can modify and potentially normalize the aberrant epigenetic information inherited at the *Dio3* locus.

**Figure 4:**
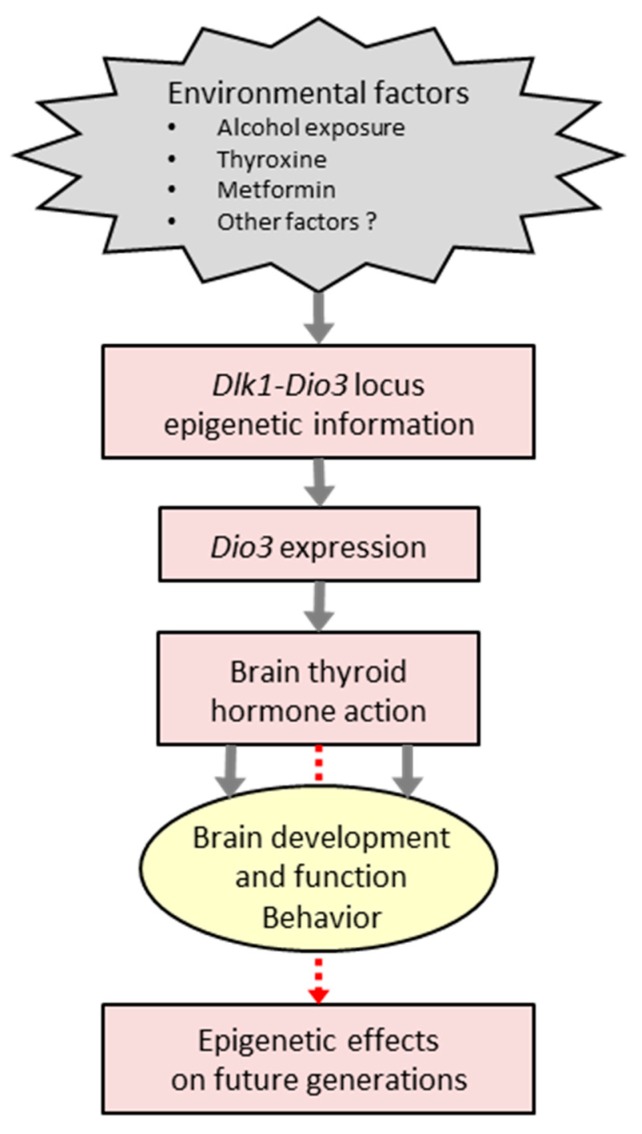
Environmental factors and *Dio3* imprinting. Environmental factors may influence *Dio3* imprinting and expression, with consequences for TH action in the brain in affected (grey arrows) and future generations (dotted red arrow).

Overall, the above work opens multiple research avenues about how environmental factors influence *Dio3* epigenetic information in current and future generations, and about the neurological traits that may be affected as a result (Figure 4).

### 5.4. Dio3 Imprinting and Brain Evolution and Adaptation

The monoallelic gene expression associated with genomic imprinting does not appear advantageous for survival, so the evolutionary reasons that supported the establishment of this phenomenon are unclear. Theories in this regard [124,125,126] are supported by some current information about genomic imprinting and the function of imprinted genes. Genomic imprinting is essentially limited to mammals [127], and imprinted gene functions typically influence the allocation of developmental resources, as they regulate placental function, fetal growth, suckling, and postnatal metabolic adaptations [77,128,129,130]. Thus, the most prevalent theory suggests that genomic imprinting may have evolved from a conflict between parental genomes, with genes from the father seeking to maximize resource usage and offspring survival, and maternal genes limiting resources to avoid compromising reproductive function while at the same time seeking the viability of all offspring regardless of paternity. Additional theories, not necessarily mutually exclusive, have also been proposed [131,132,133,134].

Genomic imprinting evolved during an evolutionary period of rapid mammalian speciation [127], which has driven speculation that environmental factors and natural selection also influenced genomic imprinting as an efficient manner of adaptation to the environment. Given the importance of THs for brain development and their unique need of iodine for synthesis, it is possible that *Dio3* imprinting was favored as a way to save iodine as mammals moved into more land-based, iodine-poor ecosystems.

The functions of many imprinted genes, including *Dio3*, also converge on the regulation of behavior [55,135]. An intriguing study in chimeric mice shows that androgenetic and parthenogenetic cells contribute very differently to brain structures. While androgenetic cells comprise most of the hypothalamus, preoptic area, septum, and bed nucleus of the stria terminalis, parthenogenetic cells drive brain size and proliferate in the striatum and cerebral cortex [136]. These observations on androgenetic cells correlate with the patterns of diencephalic expression of two imprinted genes (*Mest* and *Peg3*) that are paternally expressed [136]. This suggests that paternally expressed genes contribute to brain areas associated with neuroendocrine functions and primordial behaviors, while maternally expressed genes do so to brain regions associated with cognition. Information about *Dio3* is highly consistent with these findings based on its brain expression, imprinting patterns, and environmental susceptibility, as described in this article. As the brain evolved in different mammalian species, it is likely that the conflict between parental genomes and *Dio3* genomic imprinting also changed to achieve optimal brain function, raising the possibility that imbalances in genomic imprinting contribute to the etiology of neurological disorders in present humans [137].

Finally, a comparative analysis indicates that the genomic distance between *Dlk1* and *Dio3* is reduced in lower organisms when compared to that in birds and mammals [91]. This suggests that the two genes were already linked before the establishment of genomic imprinting in the domain, an event that coincided with the appearance in the *locus* of maternally-expressed, non-coding RNAs [91]. These RNAs exhibit predominant expression in the developing and adult brain, are specific to neurons [114], and their coordinated transcription from the maternal allele suppresses *Dio3* transcription in *cis*. Thus, reciprocal allelic expression of *Dio3* and non-coding RNA in the CNS may be driven by environmental adaptations increasing the plasticity of brain TH action in a manner that generates the optimal complement of neurological phenotypes.

## 6. Summary

The level of T3 action in the CNS needs to be restricted to a range that is adequate to the developmental stage and the biological requirements of the particular target cell or brain region. In this context, DIO3 plays a critical role in protecting neural tissue from an excessive level of T3. This is illustrated by the consequences of DIO3 loss of function in mice for brain thyroid status, patterns of brain gene expression, brain morphology, neuroendocrine and sensory functions, and mood and social behaviors. The variable genomic imprinting of *Dio3* across regions of the developing and adult brain and the environmental susceptibility of imprinted genes implicate epigenetic mechanisms in the fine-tuning of T3 action on the CNS, pointing to the *Dio3* imprinted locus as a potential mediator of environmentally-driven CNS abnormalities. Given the broad spectrum of neurological traits affected by DIO3 deficiency and their relevance to human conditions, altered *Dio3* imprinting appears as a potential epigenetic mechanism contributing to the developmental and non-genetic but heritable-etiology of neurological disorders.

## Abbreviations

THs	Thyroid hormones
CNS	Central nervous system
UPD(14)	Uniparental disomy (of chromosome 14)
IG-DMR	Intergenic differentially methylated region
